# Broad-coverage biomedical relation extraction with SemRep

**DOI:** 10.1186/s12859-020-3517-7

**Published:** 2020-05-14

**Authors:** Halil Kilicoglu, Graciela Rosemblat, Marcelo Fiszman, Dongwook Shin

**Affiliations:** 1 Lister Hill National Center for Biomedical Communications, National Library of Medicine, 8600 Rockville Pike, Bethesda, 20894 MD USA; 2 University of Illinois at Urbana-Champaign, School of Information Sciences, 501 E Daniel Street, Champaign, 61820 IL USA; 3 Independent Researcher, Rio de Janeiro, Brazil

**Keywords:** Natural language processing, Biomedical relation extraction, Semantic interpretation, Scientific publications

## Abstract

**Background:**

In the era of information overload, natural language processing (NLP) techniques are increasingly needed to support advanced biomedical information management and discovery applications. In this paper, we present an in-depth description of SemRep, an NLP system that extracts semantic relations from PubMed abstracts using linguistic principles and UMLS domain knowledge. We also evaluate SemRep on two datasets. In one evaluation, we use a manually annotated test collection and perform a comprehensive error analysis. In another evaluation, we assess SemRep’s performance on the CDR dataset, a standard benchmark corpus annotated with causal chemical-disease relationships.

**Results:**

A strict evaluation of SemRep on our manually annotated dataset yields 0.55 precision, 0.34 recall, and 0.42 F _1_ score. A relaxed evaluation, which more accurately characterizes SemRep performance, yields 0.69 precision, 0.42 recall, and 0.52 F _1_ score. An error analysis reveals named entity recognition/normalization as the largest source of errors (26.9%), followed by argument identification (14%) and trigger detection errors (12.5%). The evaluation on the CDR corpus yields 0.90 precision, 0.24 recall, and 0.38 F _1_ score. The recall and the F _1_ score increase to 0.35 and 0.50, respectively, when the evaluation on this corpus is limited to sentence-bound relationships, which represents a fairer evaluation, as SemRep operates at the sentence level.

**Conclusions:**

SemRep is a broad-coverage, interpretable, strong baseline system for extracting semantic relations from biomedical text. It also underpins SemMedDB, a literature-scale knowledge graph based on semantic relations. Through SemMedDB, SemRep has had significant impact in the scientific community, supporting a variety of clinical and translational applications, including clinical decision making, medical diagnosis, drug repurposing, literature-based discovery and hypothesis generation, and contributing to improved health outcomes. In ongoing development, we are redesigning SemRep to increase its modularity and flexibility, and addressing weaknesses identified in the error analysis.

## Background

A massive amount of biomedical knowledge is buried in free text, including scientific publications and clinical narratives. Natural language processing (NLP) techniques are increasingly used to extract from free text biomedical concepts, such as disorders, medications, tests, and genes/proteins, as well as relationships between them, including disease treatments, protein/drug interactions, and adverse drug events. Such techniques transform unstructured text into computable semantic representations, which can in turn support biomedical knowledge management and discovery applications, allowing clinicians and bench scientists to more efficiently access information and generate new knowledge.

Relation extraction from the scientific literature is a foundational task in biomedical language processing, and has been proposed as the basis of practical applications, including biological database curation [1], drug repurposing [2], and clinical decision making [3]. This task has generally been studied within the context of shared task challenges, which have considered extraction of specific relationship types, such as protein-protein interactions [4], chemical-induced disease relationships [1], causal biological network relationships [5], biological events [6–9], and drug-drug interactions [10, 11]. Benchmark corpora have been developed within the context of these shared tasks (e.g.,[1, 10, 12]) and independently (e.g., [13–15]). The majority of recent relation extraction approaches have been trained on annotated corpora using supervised machine learning techniques (e.g., [16–20]). Competitive rule-based systems have also been proposed [21–24]. More recently, deep neural network architectures using distributed representations (word, dependency and other types of embeddings) have also been proposed, often improving relation extraction performance on standard benchmarks (e.g., [25–27]). A more comprehensive survey of biomedical relation extraction from scientific literature can be found in Luo et al. [28].

### SemRep

Developed at the U.S. National Library of Medicine, SemRep [29, 30] is a broad-coverage NLP system that extracts semantic relations from biomedical text. It is a rule-based system with a strong linguistic bent; it combines syntactic and semantic principles with structured biomedical domain knowledge contained in the Unified Medical Language System (UMLS) [31, 32] to extract semantic relations. The relations extracted by SemRep are subject-predicate-object triples, also called *semantic predications*. The subject and object pair are UMLS Metathesaurus concepts with specific semantic types and the predicate is a relation type in an extended version of the UMLS Semantic Network [15]. While the primary focus of SemRep has been on research literature in PubMed, it has also been applied to clinical narratives (e.g., [33, 34]) and “gray” literature (e.g., [35]).

For an illustration of SemRep, consider the two semantic predications extracted from the input sentence in example (1). Arguments of the predications (subject and object) are represented as Concept Unique Identifier (CUI): Concept Name (Semantic Type).

1. MRI revealed a lacunar infarction in the left internal capsule.
  C0024485: Magnetic Resonance Imaging (Diagnostic Procedure)
    - diagnoses -
    C0333559: Infarction, Lacunar (Disease or Syndrome)
  C2339807: Left internal capsule (Body Part, Organ, or Organ Component)
    - location_of -
    C0333559: Infarction, Lacunar (Disease or Syndrome)

SemRep extracts a range of predicates relating to clinical medicine (e.g. TREATS, DIAGNOSES, PROCESS_OF), molecular interactions (e.g., INTERACTS_WITH, INHIBITS, STIMULATES), disease etiology (e.g., ASSOCIATED_WITH, CAUSES, PREDISPOSES), pharmacogenomics (e.g., AFFECTS, AUGMENTS, DISRUPTS), as well as static relations (ISA, PART_OF, LOCATION_OF).

The theoretical framework of SemRep, with its increased emphasis on lexical and ontological domain knowledge, has been inspired by the lexical semantics [36] and the ontological semantics [37] paradigms. SemRep also owes much to Meaning-Text Theory [38], with its notion of semantic representation as a network of predications and mapping of syntactic structures to semantic representation by rules.

The groundwork for SemRep was laid out about two decades ago, in pioneering systems such as ARBITER [39], EDGAR [40], and work on anatomic spatial relationships in clinical text [33]. Its early development was conducted in parallel with that of MetaMap [41], which SemRep continues to rely on for named entity recognition and normalization. An offshoot of SemRep, named SemGen [42, 43], focused on genetic relations (such as ASSOCIATED_WITH, STIMULATES, INHIBITS) and was supported by the ABGene gene recognition system [44] in addition to MetaMap. SemGen was later incorporated into the unified SemRep program. A major effort in the late 2000s concentrated on extending SemRep to domains under-represented in the UMLS, such as disaster information management [35], public health [45], and medical informatics [46]. Over time, SemRep has been incrementally enhanced in numerous ways, focusing on various linguistic phenomena and relation types [42, 47–50]. Its reliability and scalability have also been improved. Since 2013 (release 1.5), SemRep has been made publicly available as a standalone program (previously, it was only available through a web interface). The latest version of SemRep (release 1.8) was released in October 2018. Due partly to its roots in the PUNDIT system [51], SemRep is implemented in Prolog logic programming language. With release 1.8, we are phasing out this implementation and plan to implement future releases using Java.

Along with its use as a standalone biomedical relation extraction system, SemRep has also underpinned advanced biomedical knowledge management/discovery tools, including Semantic MEDLINE [52, 53], a Web-based application which combines SemRep processing with automatic summarization and visualization to allow the user navigate the literature through concepts and their relationships. Semantic MEDLINE and other similar tools are supported by SemMedDB [54], a publicly available PubMed-scale repository of semantic predications. In its most recent release (as of June 30, 2019), SemMedDB contains about 98 million predications from over 29 million PubMed abstracts.

In this paper, our objective is two-fold. First, we aim to address a gap by providing an up-to-date, in-depth description of the SemRep pipeline (release 1.8). While various aspects of SemRep processing have been reported and evaluated over the years [29, 30, 42, 47–50], a complete overview and a comprehensive evaluation of the system has not been previously reported. Our second goal is to present a qualitative assessment of SemRep, by comparing it to other relation extraction systems, illustrating its broader impact on downstream applications, and discussing future directions.

## Implementation

In this section, we present the steps of the SemRep pipeline, with minimal examples for illustration. The interpretation of a full sentence, taken from the PubMed abstract 12975721, with the corresponding pipeline steps is provided as supplementary material in Additional file 1.

**Fig. 1:**
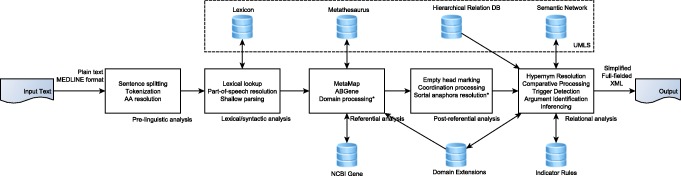
High-level overview of the SemRep pipeline. Processes marked with * are optional (*domain processing* and *sortal anaphora resolution*).

The SemRep pipeline can be broken down into five broad analysis steps, illustrated in Fig. 1: *pre-linguistic analysis*, *lexical/syntactic analysis*, *referential analysis*, *post-referential analysis*, and *relational analysis*. Each of these steps consist of several specific tasks, discussed below. First, we briefly touch upon SemRep input and output.

### Input and output

SemRep takes as input ASCII-formatted plain text or text in PubMed’s MEDLINE format. The output is made available in several formats:

- The *simplified plain text format* consists of sentences and the predications extracted from them, as presented in example (1).
- The *full-fielded format* is more verbose, and consists of all entities as well as predications along with the sentences they are extracted from. We include more information about sentences, entities, predications, and (optionally) coreference relations. Such information includes character offsets, entity confidence scores, and predication indicator types (see https://github.com/lhncbc/SemRep/blob/master/doc/SemRep_full_ fielded_output.pdffor details).
- The *XML format* presents an XML representation of the full-fielded output format (see https://github.com/lhncbc/SemRep/blob/master/doc/SemRep.v1.8_XML_ output_desc.txtfor details).

### Pre-linguistic analysis

The first step in SemRep processing, pre-linguistic analysis, consists of sentence splitting, tokenization, and acronym/abbreviation detection. For the MEDLINE-formatted input text, we also identify the PubMed ID, title, and abstract portions of the text. SemRep relies entirely on MetaMap functionality to perform the pre-linguistic analysis tasks. It is worth noting that the acronym/abbreviation detection algorithm used by MetaMap is an adaptation of the algorithm proposed by Schwartz and Hearst [55], which matches a bracketed acronym/abbreviation with a potential expansion that precedes it in the same sentence. SemRep tokenization treats hyphens and parentheses as individual tokens. For example, the string *beta1-adrenergic receptor (beta1AR)* is tokenized as follows, and *beta1AR* is recognized as the acronym for *beta1-adrenergic receptor*.

- [beta1, -, adrenergic, receptor, (, beta1AR,) ]

The unit of processing for SemRep is the sentence. All the subsequent steps operate on one sentence at a time.

### Lexical/syntactic analysis

A lookup to the UMLS SPECIALIST Lexicon [56] provides lexical and syntactic information about tokens identified in the pre-linguistic analysis. Such information includes lemma, part-of-speech tags, subcategorization frames, grammatical number (singular, plural), as well as inflectional and derivational variant information. Lexical lookup also identifies some multi-word expressions. For illustration, lexical entries retrieved for the verb *reduced* and the multi-word expression *calcium antagonists* are presented in Table 1. The entry for *reduced* indicates that the lemma (*base*) of the verb is *reduce*, its generalized part-of-speech (*cat*) is verb, that *reduced* is a regular inflectional variant of the verb *reduce*, that it can be used intransitively as well as transitively (e.g., attaching to a prepositional phrase (*pphr*) with the cue *to*), and that it has two nominalized forms *reduction* and *reducement*.

**Table 1:** Lexical entries retrieved for *reduced* and *calcium antagonists*

input=reduced	input=calcium antagonists
base=reduce	base=calcium antagonist
entry=E0052363	entry=E0515276
cat=verb	cat=noun
variants=reg	variants=reg
intran	variants=uncount
tran=np	spelling_variant=calcium-antagonist
tran=pphr(from,np,pphr(to,np))	number=plural
tran=pphr(to,np)	
ditran=np,pphr(from,np,pphr(to,np))	
ditran=np,pphr(to,np |*tears*|)	
cplxtran=np,pphr(to,ingcomp:objc)	
nominalization=reduction |*noun*|E0052372	
nominalization=reducement |*noun*|E0525409	
tense=past,pastpart	

Lexical lookup may reveal part-of-speech ambiguities, with multiple entries returned for a given lexical unit. For example, two lexical entries are retrieved for *have*, one in which the part-of-speech is auxiliary and one in which it is verb. In such cases, we consult the MedPost part-of-speech tagger [57] for disambiguation.

Information retrieved from the SPECIALIST Lexicon and the MedPost Tagger is used by our shallow parser (named *minimal commitment parser*) to generate a partial syntactic analysis by identifying simple noun phrases (i.e., those with no post-modification) and their internal structure (head and modifiers). Shallow parsing is based on the notion of *barrier words*, which open a new phrase and close the preceding one. Verbs, prepositions, conjunctions, modal auxiliaries, and complementizers are marked as barrier words. Any phrase containing a noun is considered to be a simple noun phrase (henceforth referred to as NP), and the right-most noun is labeled as the head. All other items except determiners are labeled as modifiers. An NP whose first element is a preposition is treated as a prepositional phrase^1^. Other syntactic categories, including verbs and conjunctions, are simply given their part-of-speech label and treated as separate phrases.

The lexical/syntactic analysis step is also shared between MetaMap and SemRep.

### Referential analysis

Referential analysis is the process of identifying named entity mentions in text and mapping them to the corresponding ontological concepts. Currently, this analysis consists of three steps (one of them optional):

- Using MetaMap to map NPs to UMLS Metathesaurus concepts
- Using ABGene to identify gene/protein mentions and normalizing them to NCBI Gene [58] identifiers
- Using domain extensions to recognize additional concepts or suppress identified concepts (optional) (more below)

#### MetaMap

The UMLS Metathesaurus is the main source of terminological knowledge in SemRep. MetaMap [41] is used to map NPs identified with lexical/syntactic analysis to UMLS Metathesaurus concepts, with their concept unique identifiers (CUIs), preferred names, and semantic types (see Aronson and Lang [41] for a general overview of MetaMap). MetaMap usage in SemRep diverges from the default behavior of MetaMap as follows:

- We use MetaMap with the 2006AA UMLS Metathesaurus USABase dataset by default, due to the prevalence of concept ambiguity in the later UMLS releases [41] and SemRep’s optimized conceptual and relational modifications for said release (though, the most recent UMLS dataset is available as an option).
- We use the word sense disambiguation option of MetaMap, with the *semantic type indexing* method for disambiguation [59].
- We rely on the NegEx [60] algorithm as implemented in MetaMap to recognize negated mentions, but we use a narrower window size than MetaMap for negation (within a window of 2 concepts). We also use a customized negation trigger list for biomedical literature (354 triggers, including fail to and no evidence) and apply NegEx processing to all semantic types^2^.
- We suppress some mappings identified by MetaMap to account for spurious ambiguity in the UMLS Metathesaurus. We start by blocking spurious Metathesaurus synonyms, which we name *dysonyms*, from being considered by MetaMap in candidate mapping evaluation. Dysonyms are only truly synonymous with a specific UMLS concept in a limited domain covered by one of the constituent UMLS terminologies, but are not valid broadly. We identify dysonyms by considering substring relationship between the synonym and the preferred name of the corresponding UMLS concept. For example, in the Metathesaurus, influenza is a synonym of the concept C0021403: Influenza virus vaccine, in addition to being a synonym of the concept C0021400: Influenza. The validity of the former is limited to specific contexts discussing the vaccine. The synonym influenza is a substring of the preferred name Influenza virus vaccine, so it is taken as a dysonym with respect to this concept. Thus, the concept C0021403: Influenza virus vaccine is blocked from being used as a mapping for the string *influenza*. There are some exceptions to dysonym processing. Some synonyms are allowed even though they satisfy the substring constraint, because the remaining part of the preferred name consists of a general term which does not invalidate the mapping. Such terms include *procedure*, *disorder*, or *gene*. In addition to substring processing, we maintain a list of dysonyms that do not satisfy the substring constraint. Our current list includes 706 such items that allow us to block mappings such as *best* mapping to C0339510: Vitelliform dystrophy or *favor* to C0309050: FAVOR, a supplement brand name.

#### ABGene

NCBI Gene database [58] serves as a supplementary source to the UMLS Metathesaurus with respect to gene/protein terms, as the Metathesaurus coverage for these terms is not exhaustive. In SemRep, we recognize gene/protein mentions using ABGene [44] in addition to MetaMap. Mapping to NCBI Gene identifiers is facilitated by a pre-computed index, in which gene aliases and the corresponding official symbols (and their identifiers) in NCBI Gene are used as key-value pairs. This index is currently limited to human genes/proteins. We use exact matching criterion between the mention and a gene alias to map mentions identified by ABGene and MetaMap to NCBI Gene identifiers. The identified NCBI Gene term is assigned the semantic type Gene or Genome. A mention can be mapped to several NCBI Gene terms. We do not perform disambiguation on these terms and simply provide all NCBI Gene terms identified through exact matching. We do not distinguish between genes and the gene products (proteins) using the same symbol, in line with most other NLP systems. In the text snippet *Ataxin-10 interacts with O-GlcNAc transferase OGT* below, *Ataxin-10* is mapped to both UMLS Metathesaurus and NCBI Gene and *OGT* only to NCBI Gene.

- *Ataxin-10* →C1538308: ATXN10 gene |25814: ATXN10(Gene or Genome)
- *OGT* →8473: OGT (Gene or Genome)

#### Domain extensions

Domain extensions to SemRep enable extraction of semantic relations in specific domains under-represented in the UMLS (e.g., disaster information management [35]). These extensions were later incorporated into unified SemRep as processing options (e.g., –domain disaster for disaster information management).

A domain extension is formalized as a set of Prolog statements about concepts and relations in a new domain (see Rosemblat et al. [46] for a comprehensive discussion). Briefly, four types of terminological extensions are formalized as presented below, with illustrative examples from the disaster information management domain.

- Semantic types relevant to the domain (e.g., Community Characteristics)
- Domain-inappropriate UMLS mappings to block (e.g., *board* →C0972401: Boards (Medical Device))
- Recontextualized UMLS concepts (e.g., C0205848: Death Rate (Quantitative Concept) recontextualized as C0205848: Death Rate (Community Characteristics))
- New domain concepts and their synonyms (e.g., D0000233: Health Alert Notice (Information Construct) with synonyms health alert and health alert notice)

These terminological extensions are applied as the last step of the referential analysis. Extensions related to domain relationships, relevant in the relational analysis step, are discussed in later sections.

Based on the domain extension formalization, beginning with the 1.8 release, we provide two additional options to customize the generic SemRep processing for increased coverage. The *generic domain extension* option (-N) allows SemRep to use an extended set of concepts, while the *generic domain modification* (-n) allows recontextualizing existing UMLS concepts. An example in the extended concept set is G0000211: cancer-free survival (Organism Function) with the synonym cancer-free survival, a common outcome measurement with no corresponding concept in the UMLS Metathesaurus. An example of a recontextualized UMLS concept is C0337664: Smoker, whose semantic type is changed from Finding to Population Group/Human. These extensions, implemented through manual analysis of SemRep results over the years, aim to address UMLS Metathesaurus limitations and to increase SemRep precision/recall. The extended concept set currently consists of 588 new concepts and 336 recontextualized UMLS concepts.

### Post-referential analysis

Referential analysis is followed by empty head marking, coordination processing, and optionally, sortal anaphora resolution. These steps expand the scope and specificity of relational analysis (see next section) by filtering out semantically empty words/phrases and establishing semantic dependencies between NPs.

#### Empty head marking

SemRep considers the head of a NP its most salient semantic element, and the relational analysis relies heavily on the semantics of the head. A common feature in the biomedical literature is that NP heads can be semantically empty with respect to the UMLS Metathesaurus, as they can be generic expressions with a non-informative semantic type. Such nouns are sometimes referred to as *empty heads* [61]. For example, in the clause *activation of CYP2C9 variants by dapsone*, the head of the NP *CYP2C9 variants* (i.e., *variants*) is considered an empty head as it is mapped to a concept with the uninformative semantic type Qualitative Concept. In such cases, the most salient element of the phrase is generally the modifier preceding the empty head. We maintain a list of empty head nouns in SemRep (241 nouns), and adjust the syntactic analysis when a NP is headed by an empty head. In these cases, the first modifier to the left of the empty head (*CYP2C9* in the example above) is relabeled as the semantic head of the NP. In addition to genetic phenomena (such as *variant*, *polymorphism*), this list includes measurement- (e.g. *concentration*) and process-related words (e.g., *synthesis, metabolism*).

#### Coordination processing

SemRep performs limited coordination processing, focusing primarily on NP coordination. The process first determines whether each coordinating conjunction (e.g., *and*, *or*) conjoins NPs. Several multi-word expressions (*followed by*, *in combination with*, *but not*) are also treated as coordinating conjunctions. Conjunctions preceding coordinate NPs (e.g., *either*, *both*) are ignored.

For a conjunction that conjoins NPs, we check whether the NPs before and after the conjunction are compatible (i.e., they are conjuncts). Two NPs are compatible only if one of the following conditions apply:

- They are semantically compatible. The semantic types associated with their semantic heads belong to the same semantic group [62] in the UMLS Semantic Network (i.e., coarse-grained semantic classes, such as Disorders or Drugs & Chemicals).
- They have the same head word.
- They are both relational nouns. SemRep currently uses a list of 151 relational nouns, which includes *application*, *analysis*, and *synthesis*.

If the NPs to the left and to the right of the conjunction are conjuncts, we try to detect series coordination by repeating the process for NPs occurring further to the left of the left NP and separated from it by a comma. This process is terminated when an incompatible NP or a barrier word is encountered. Barrier words in this case include *between, either, against, such as, including*.

In the snippet *osteosarcoma, melanoma, and breast cancer*, SemRep is able to recognize that the NPs *osteosarcoma*, *melanoma*, and *breast cancer* are conjuncts, as they are semantically compatible (all belong to Disorders semantic group) and are separated by the coordinating conjunction *and* and commas.

We currently do not address more complex cases of coordination, such as verbal/clausal coordination (e.g., *Infections can trigger GBS and exacerbate CIDP.*) and coordination ellipsis (e.g., *the male and the female genital tract*).

#### Sortal anaphora resolution

Coreference resolution is the task of identifying textual expressions referring to the same real-word entity [63]. *Sortal anaphora* (also called nominal anaphora) is a type of coreference indicated by a NP (*anaphor*), which refers to a previously mentioned entity (*antecedent*). An example of sortal anaphora can be the NP *this disease* (anaphor) referring to *diabetes* (antecedent) mentioned earlier in the discourse. Resolution of sortal anaphora is optional in SemRep and, when used, not only can it increase the specificity of the generated relations, but it can also expand the scope of relation extraction beyond the sentence level.

Sortal anaphora resolution in SemRep and its effect on relation extraction is discussed in depth in Kilicoglu et al. [50]. Briefly, this process consists of two steps: anaphor detection and linking of anaphors to their corresponding antecedents. In the first step, candidate anaphoric NPs are recognized based on whether they contain a determiner or an adjective that can indicate a sortal anaphor (e.g., *these*, *each*, *such*). These phrases are then checked for anaphoricity, and non-anaphoric phrases are filtered out. One anaphoricity filter ensures that the candidate NP is not in an appositive construction. For example, in the clause *the gene, BRCA1, is…*, *the gene* is non-anaphoric because it is in an appositive structure. Linking of anaphors to their antecedents relies on semantic compatibility and grammatical number agreement. One semantic compatibility constraint relies on taxonomic relations between UMLS Metathesaurus concepts, and requires that the concept associated with the anaphor (A) be an ancestor of the concept associated with the candidate antecedent (B). For example, this constraint predicts that the NP *cetirizine* (B) can be an antecedent for the anaphor *this drug* (A). The anaphor and the antecedent are also required to have number agreement (both singular or both plural). Sortal anaphora resolution accounts for coordination, potentially linking a sortal anaphor like *these drugs* to several coordinate NPs as in the snippet *low-dose diuretics, beta-blockers, and dihydropyridine calcium antagonists*.

Pronominal anaphora (e.g., the pronoun *it* referring to the drug *cetirizine*) is less frequent in biomedical literature [64] and is currently unaddressed in SemRep.

### Relational analysis

Relational analysis is the process of predication generation based on lexical, syntactic and semantic knowledge collected in the previous steps. Two types of predications, hypernymic predications (i.e., ISA) and comparative predications (e.g., HIGHER_THAN), are generated through specialized machinery [29, 48]. All other associative predications are generated using a uniform trigger detection and argument identification mechanism. The final step of relational analysis is inferencing, in which generated predications form the basis for generating additional, more specific predications. These steps are described below. For brevity, we generally omit concept identifiers or semantic types in the examples.

#### Hypernym resolution

A hypernymic predication involves two concepts in a taxonomic (ISA) relationship, the subject argument semantically more specific (*hyponym*) and the object more general (*hypernym*). The generation of such predications in SemRep is discussed in detail in Rindflesch and Fiszman [29].

In short, SemRep focuses on three syntactic manifestations of such predications:

- Nominal modification: The head and the modifier of a NP correspond to a candidate hyponym/hypernym pair (e.g., *the anticonvulsantgabapentin*).
- Appositive structures: Two NPs in an appositive construction contain the candidate pair (e.g., *Non-steroidal anti-inflammatory drugs such as indomethacin*)
- Verbal triggers: Two NPs separated by one of two verbs (*be* or *remain*) and within a pre-specified window size of each other (5 phrases) contain the candidate pair (e.g., *Modafinil is a novel stimulant …*)

After a candidate pair has been identified, regardless of the structure, it is subjected to UMLS-based semantic constraints. First, we require that the concepts of the pair be in the same semantic group. Concepts in two specific semantic groups (Anatomy and Concepts & Ideas) are excluded from consideration in this step; the former because the UMLS hierarchy includes some meronymic relations (PART-OF) [65] that can interfere with hypernymy processing and the latter because it is too heterogeneous with respect to the semantic types it contains to be useful (e.g., Temporal Concept and Group Attribute). The second constraint is that the concepts must be in a hierarchical relationship in the UMLS Metathesaurus concept hierarchy.

Based on the constraints, SemRep generates the predication gabapentin-ISA-Anticonvulsants from the snippet *the anticonvulsant gabapentin*.

#### Comparative processing

SemRep focuses on interpretation of two types of comparative structures, one in which a comparison is simply stated in the text, as in Example (2) below, and the other in which the relative ranking of two compared terms on a scale is also indicated (Example (3)). For both types, SemRep generates a COMPARED_WITH predication. For the second type, it also generates a predication indicating the relative value on the scale (HIGHER_THAN, LOWER_THAN, or SAME_AS), as well as the name of the scale that is the basis for comparison. The scale in this example is identified as the EFFECTIVENESS scale, based on the cue *effective*.

(2) To compare misoprostol with dinoprostone for cervical ripening …
  Misoprostol-compared_with-Dinoprostone
(3) Amoxicillin-clavulanate was not as effective as ciprofloxacin for treating uncomplicated bladder infection ….
  Amoxicillin-Potassium Clavulanate Combination-compared_with-Ciprofloxacin
  Amoxicillin-Potassium Clavulanate Combination-lower_than-Ciprofloxacin

The process for generating comparative predications is detailed in Fiszman et al. [48]. Briefly, two sets of lexico-syntactic patterns are used, one for each type of comparative structures. For example, the pattern <comparison of Term1 with/to Term2 > identifies a construction of the first type, while <Term1 BE as ADJ as {BE} Term2 > addresses the second type of construction, in which BE indicates a form of the verb *be*, and {BE} indicates that this verb is optional. The patterns are recognized using the syntactic structure already identified. In addition, semantic compatibility constraints are applied to Term1 and Term2, as in hypernymy and coordination processing. Comparative processing was initially limited to interventions and it was later expanded to apply to all semantic groups.

#### SemRep relation ontology

Before describing the generation of associative predications, it is important to briefly discuss the SemRep relation ontology, as it is an essential resource underlying the rest of the steps. The SemRep ontology is an extension of the UMLS Semantic Network, and serves as an upper-level domain model consisting of predicate types (e.g., TREATS) and the relationships that can hold between semantic types (i.e., *ontological predications*). An example ontological predication is Pharmacologic Substance-TREATS-Disease or Syndrome.

In the SemRep ontology, we use a subset of the 55 relations in the UMLS Semantic Network. We redefined five relations (ASSOCIATED_WITH, DISRUPTS, INTERACTS_WITH, OCCURS_IN, PROCESS_OF), added seven new relations (ADMINISTERED_TO, AUGMENTS, COEXISTS_WITH, CONVERTS_TO, INHIBITS, PREDISPOSES, STIMULATES), and expanded 13 relations with respect to their ontological predications (AFFECTS, CAUSES, COMPLICATES, DIAGNOSES, LOCATION_OF, MEASURES, METHOD_OF, PART_OF, PRECEDES, PREVENTS, PRODUCES, TREATS, USES), while excluding 30 relations (e.g., ANALYZES, ADJACENT_TO, BRANCH_OF). In all, 25 relations (excluding ISA and comparative predicates) are used in the SemRep ontology. For descriptions of all predicates and examples in which they apply, see the Appendix in Kilicoglu et al. [15].

SemRep ontology defines semantic constraints on arguments and, thus, it plays a central role in linking a predicate to its arguments. In this process, ontological predications from the original UMLS Semantic Network are considered first, followed by those in a supplementary ontology manually developed over time. Currently, we use a total of 7398 ontological predications: 3100 (41.9%) from the UMLS Semantic Network and the rest (4298 - 58.1%) from the supplementary ontology. A full list of ontological predications in the SemRep ontology is provided as supplementary material in Additional file 2.

Each domain extension of SemRep defines its own supplementary ontology to be used to augment the UMLS Semantic Network. For example, the disaster information management extension defines 14 predicate types (e.g., ALERTS) and 556 ontological predications (e.g., Organization-MONITORS-Virus).

#### Trigger detection with indicator rules

Excluding hypernymic and comparative predications, generation of other types of predications begins with the detection of lexical elements and syntactic structures that trigger particular predicate types. This is achieved using *indicator rules*, each of which maps a lexical entry (with a specific part-of-speech tag and, optionally, an argument cue) to one of the 25 predicates that SemRep uses. Some indicator rules are structural rather than lexical, mapping the modifier-head structure in an NP to a predicate [66]. Lexical elements currently included in indicator rules are verbs, nominalizations and other relational nouns (including gerunds), prepositions, and adjectives. Argument cues are only relevant for verbs and nouns, and are used to place syntactic restrictions on the arguments that the predicate can take. Two example indicator rules are given below (in the form of LexicalItem:PartOfSpeech:Cue(Argument) →PREDICATE):

- treat:verb:none →treats
- treatment:noun:with(subject) →treats

The first rule indicates that a token with the lemma *treat*, when tagged as a verb (e.g., *treated, treats*), triggers the predicate TREATS. The fact that there is no Cue element (none) indicates that the arguments of the verb should not be cued by a preposition (i.e., they can be in an NP). This rule would be fired for the snippet *Aspirin treats headache*. The second rule indicates that the nominalization *treatment* can trigger the predicate TREATS, provided that a subject argument can be found in a prepositional phrase introduced by *with*. This rule would be triggered for the snippet *treatment of headache with aspirin*. One modifier-head indicator rule involves the PROCESS_OF predicate, and would be triggered for the NP *diabetic patients*.

A small number of indicator rules involve more complex phrasal and clausal elements, such as increased risk and {increase,odds}, both with the object cue for, corresponding to the predicate PREDISPOSES. In the latter, the comma indicates that determiners or other modifiers are allowed between the trigger words (e.g., *increase the odds*).

SemRep currently uses a total of 1366 indicator rules: 1256 consist of a single word, 105 based on phrases and clausal elements, and 5 based on the modifier-head structure. INTERACTS_WITH is the predicate with the highest number of indicator rules (195) and MEASURES the one with the lowest (6). A full list of indicator rules is provided as supplementary material in Additional file 3.

Domain extensions in SemRep also incorporate a set of indicator rules. Two indicator rules from the disaster information management domain are:

- caution:verb:none →alerts
- contamination:noun:none →infects

#### Argument identification

SemRep ontology and indicator rules in conjunction with the syntactic/semantic knowledge associated with phrases underpin argument identification. Different syntactic argument identification rules are triggered based on the class of the indicator (verb, preposition, etc.). Other constraints apply broadly. For example, one constraint limits the use of an argument in multiple predications (*argument reuse* below). The arguments of a predicate are not allowed to be conjuncts unless the triggering indicator rule has the argument cue between-and. Most importantly, the predication generated by the argument identification process must be licensed by an ontological predication in the SemRep ontology. Below, we briefly describe and exemplify the syntactic rules. These rules also apply in domain extensions without any modifications.

##### Verbal indicator rules

Syntactic argument identification rules for verbal indicators stipulate that the subject argument must occur to the left of the verb and the object to the right. If a verb is recognized as being in passive voice, the order of its arguments is reversed. If the indicator rule being applied specifies an argument cue, we require that the argument be in a prepositional phrase marked by that cue. In the example below, Urinary tract infection (Disease or Syndrome) is recognized as the subject argument and Pyelonephritis (Disease or Syndrome) as the object, due to the indicator rule and the ontological predication below.

(4) …pyelonephritis in cattle most commonly **result from** ascending urinary tractinfection
  Indicator rule: result:verb:from(subject) →causes
  Ontological predication: Disease or Syndrome-causes-Disease or Syndrome
  SemRep output: Urinary tract infection-causes-Pyelonephritis

##### Prepositional indicator rules

The primary constraint for prepositional indicators is that the subject be to its left, with the object being in the NP introduced by the preposition. Two other constraints are aimed at more precise recognition of the subject arguments [67]. One uses subcategorization information from the lexical lookup so only those prepositions not subcategorized for by the head word preceding the preposition can act as triggers. The other constraint limits the subject argument of prepositions of, for, from, and with to the preceding NP. An example of a predication generated due to a prepositional indicator rule is:

(5) …vertical banded gastroplasty**for**morbid obesity
  Indicator rule: for:prep:none →treats
  Ontological predication: Therapeutic or Preventive Procedure-treats-Disease or Syndrome
  SemRep output: Vertical-Banded Gastroplasty-treats-Obesity, Morbid

##### Nominal indicator rules

Syntactic constraints that apply to nominalizations and other argument-taking nouns (e.g., treatment and therapy, respectively) are significantly more complex and are based on 14 nominal alternation patterns identified in prior work [49]. These patterns include one in which both arguments are to the right of the indicator (*treatment of fracture with surgery*) and another in which both arguments precede the indicator as modifiers (*surgical fracture treatment*). Syntactic constraints based on these alternation patterns consider the position of the arguments with respect to each other and to the nominal trigger, and whether they modify the trigger or not (see Kilicoglu et al. [49] for details). A few points are worth repeating here. First, syntactic constraints for nominal triggers consider not only prepositional cues specified in the indicator rules but also verbs (most commonly a form of be), comma, or parenthesis as cues. Second, verbs, comma, parenthesis, and the prepositions by, with, and via act as cues for subject arguments only. Third, the preposition of acts as a cue for subjects only if the trigger has an obligatory object cue (e.g., *the contribution of stem cells to kidney repair* where *to* is an obligatory object cue for *contribution*). Lastly, a class of nominal indicators (e.g., cause) do not allow a prepositionally cued subject. An example is given below.

(6) *…the ****contribution**** of stem cells to *kidney repair
  Indicator rule: contribution:noun:to(object) →affects
  Ontological Predication: Cell-affects-Organism Function
  SemRep output: Stem cells-affects-Wound healing

##### Adjectival indicator rules

Syntactic constraints for adjectival indicators are largely similar to those for verbs, except for hyphenated adjectives, for which the subject and object arguments are required to be in the same phrase as the indicator, to its left and to its right, respectively [67].

(7) *ErbB2*-***mediated****tumorigenesis*
  Indicator rule: mediated:adj:none →affects
  Ontological predication: Gene or Genome-affects-Neoplastic Process
  SemRep output: ERBB2 Gene-affects-Tumorigenesis

##### Argument reuse

A broadly applicable syntactic constraint concerns argument reuse, which stipulates that no argument can be used in the interpretation of more than one predication without license. Two licensing phenomena are accounted for: coordination and relativization. With respect to coordination, if a conjoined NP is found to be an argument of a semantic predicate, then all NPs conjoined with that NP must also be arguments of a predication with that predicate. In the example below, *pyelonephritis* is coordinated with *cystitis* and *urethritis*. For this reason, in addition to Urinary tract infection-CAUSES-Pyelonephritis, two additional predications are generated, illustrating the reuse of the subject argument Urinary tract infection due to NP coordination.

(8) *Cystitis, urethritis and pyelonephritis in cattle most commonly result from ascending urinary tract infection …*
  Urinary tract infection-causes-Pyelonephritis
  Urinary tract infection-causes-Cystitis
  Urinary tract infection-causes-Urethritis

Heads of relative clauses are also allowed to be used in more than one predication. The syntactic structure identified by SemRep does not explicitly mark relative clauses. As an approximation, we recognize the head of a relative clause when it precedes an overt relativizer (such as *which*) or when it precedes a prepositional phrase, of which it is an argument (a *reduced relative clause*). This licensing rule allows construction of the first CAUSES predication from the example above (Urinary tract infection-CAUSES-Pyelonephritis). This is because the predication in (9) below has already been generated from this snippet; the preposition *in* acts as the indicator and is immediately to the right of the NP *pyelonephritis*, the reduced relative clause head.

(9) Pyelonephritis-process_of-Cattle

#### Negation processing

Once the arguments of a semantic predicate are identified, we check whether the predicate or either of the arguments is negated. If so, a negated counterpart of the predication is generated (e.g., Aspirin-NEG_TREATS-Headache, instead of Aspirin-TREATS-Headache). To recognize negation of arguments, we rely on NegEx machinery in MetaMap, with customizations (described earlier).

For the negation of predicates, several rules have been implemented. One is restricted to predications generated from modifier-head structures. We look for the prefix *non-* before the modifier in such cases, and if found, we generate a negated predication. For example, in *non-diabetic patients*, the generated predication is Diabetes-NEG_PROCESS_OF-Patients.

When the arguments are from different NPs, the process is more involved. We begin by marking triggers that may indicate predicate negation. These include not, neither, no, without, unable, and failure. Some of these triggers do not indicate negation (*pseudo-negation*) when they are followed or preceded by particular words (e.g., not only, not necessarily, without doubt, and no more than). We exclude pseudo-negation from consideration. For each predicate, we check whether it is in the scope of a negation trigger. A predicate is in the scope of a negation trigger if it immediately follows the trigger or the tokens between the predicate and the negation trigger are adverbs or part of a verbal complex (i.e., they have the part-of-speech tag modal, verb, or auxiliary). If this constraint is satisfied, a negated predication is generated. In the example below, the negation trigger is *not*.

(10) Overnight incubation with 1 microM safrole did not **alter**cell proliferation
  Indicator rule: alter:verb,none →affects
  SemRep output: Safrole-neg_affects-Cell Proliferation

It is also worth noting that some indicator rules accommodate negation implicitly. For example, the verb lack is directly mapped to several negated predicates (NEG_PROCESS_OF, NEG_PART_OF, among others). If such an indicator is negated in text (as in *did not lack*), a positive predication gets generated (PROCESS_OF instead of NEG_PROCESS_OF).

#### Incorporating sortal anaphora resolution with predication generation

In the discussion of argument reuse above, we illustrated how coordination can lead to the generation of additional predications. Similarly, when used as an option, sortal anaphora resolution can lead to the construction of additional predications. It can also lead to a more specific predication than originally generated. In the simple case, if one of the identified arguments corresponds to an anaphoric expression, the resulting predication will have the antecedent in the same argument position. If the anaphora is a case of set-membership anaphora, we generate multiple predications, with each antecedent occupying the same argument position in a different predication [50]. In the example presented below, without anaphora resolution we only generate the predication Pharmaceutical Preparations-TREATS-Pulmonary arterial hypertension in the second sentence. With anaphora resolution, this predication is substituted by three more specific, cross-sentence predications.

(11) *There are currently 3 classes of drugs approved for the treatment of PAH: prostacyclin analogues, endothelin receptor antagonists, and phosphodiesterase type 5 inhibitors…the current evidence supports the long-term use of these drugs for the ****treatment**** of patients with PAH*.
  Before: Pharmaceutical Preparations-treats-Pulmonary arterial hypertension
  After: Epoprostenol-treats-Pulmonary arterial hypertension
    After: Endothelin receptor antagonist-treats-Pulmonary arterial hypertension
    After: Phosphodiesterase 5 inhibitor-treats-Pulmonary arterial hypertension

#### Inferencing

The final step in relational analysis is drawing inferences based on generated predications. Inferencing is based on a set of rules that combine two predications into a single more specific one, increasing expressivity of predications and potentially their usefulness. These rules are applied at the sentence level. There are currently 13 inference rules. The rules are implemented in the form of IF <*premise*> THEN <*conclusion*> rules. The premise is stated as a pair of generated predications and the conclusion as a new predication. An example is given below, with the predications generated with inferencing marked as such (INFER).

(12) *replacement arthroplasty for adults with an extracapsular hip fracture*
  Rule: IF <X-TREATS-Y AND Z-PROCESS_OF-Y > THEN <X-TREATS-Z >
  Premise1: Hip Fractures-process_of-Adult
  Premise2: Arthroplasty, Replacement-treats-Adult
  Conclusion: Arthroplasty, Replacement-treats(infer)-Hip Fractures

## Results

In this section, we first briefly discuss prior focused evaluations of SemRep. Next, we present two new evaluations of SemRep performance, one using the SemRep test collection [15] and the other using the CDR corpus [1].

### Prior evaluations

Some of prior SemRep evaluations were intrinsic, focusing on SemRep performance on a specific linguistic structure (e.g., comparative predications [48]) or a specific domain of predications (e.g., pharmacogenomics [47]). With the considerable difficulty of generating a gold standard of semantic predications based on the UMLS domain knowledge, some of these intrinsic evaluations focused only on precision, while others considered both precision and recall. We present a summary of these evaluations, along with citations to the corresponding studies, in Table 2.

**Table 2:** Results of prior intrinsic SemRep evaluations

**Evaluation type**	**# Sentences**	**# Predications**	**Precision**	**Recall**
Gene-disease relations [42]	1000	1124	0.76	-
Pharmacogenomic relations [47]	300	850	0.73	0.55
Hypernymic relations [29]	-	830	0.83	-
Comparative structures [48]	287	288	0.96	0.70
Nominal predications [49]	300	300	0.75	0.57
Substance interactions [73]	200	489	0.59	0.44
Gene-function relations [73]	100	200	0.65	0.42

SemRep has also been extrinsically evaluated for its contribution to downstream tasks. These tasks include automatic summarization [68–70], ranking drug interventions for diseases [71], drug indication extraction [72], discovery of drug-drug interactions in clinical data [73], and question answering [74].

### Evaluation on the SemRep test collection

In this study, we used the SemRep test collection [15] for a broad performance evaluation of SemRep release 1.8. The SemRep test collection consists of 1371 semantic predications from 500 sentences randomly selected from 308 PubMed abstracts on a wide range of topics. We used the default processing options of SemRep, and calculated precision, recall, and F _1_ score as evaluation metrics.

The results of this evaluation are given in Table 3. In strict evaluation, in which a perfect match of concepts and predicates was required for a true positive predication, SemRep yielded 0.55 precision, 0.34 recall, and 0.42 F _1_ score. We noted that in some cases strict evaluation overpenalized SemRep or that the test collection had problems (i.e., missing predications or incorrect annotations). The relaxed evaluation, which takes these issues into account, yielded 0.69 precision, 0.42 recall, and 0.52 F _1_ score. We consider the relaxed evaluation as a more accurate characterization of SemRep performance.

**Table 3:** SemRep 1.8 evaluation against the test collection

	Precision	Recall	F _1_
Strict evaluation	0.55	0.34	0.42
Relaxed evaluation	0.69	0.42	0.52

Relaxed evaluation allows interchangeable concepts and ignores test collection annotation errors

We also analyzed the errors that SemRep made (false positives and false negatives) and categorized them according to their root causes. In brief, we found that most errors occurred in the relational analysis steps (51.5%). On the other hand, MetaMap processing was the subcategory that accounted for the highest number of errors (26.9%). More details about the error analysis and relevant examples are provided as supplementary material in Additional file 1.

### Evaluation on the CDR corpus

In the second evaluation, we assessed SemRep on a standard benchmark corpus. We considered the CDR corpus [1], developed for the BioCreative V CID task and manually annotated for chemical-induced disease relationships. We used the test set portion of this corpus, which consists of 500 abstracts. Each abstract in the corpus is annotated with chemical and disease mentions normalized to MeSH identifiers. Causal relationships between normalized chemical-disease pairs are annotated at the abstract level. No relation triggers are annotated. In 27.2% of the relationships, entity pairs do not co-occur in the same sentence of the abstract (i.e., they are cross-sentence relationships) [75]. In addition to measuring SemRep performance on the entire CDR test set (SemRep-ALL, 1066 ground truth relationships), we also measured it limiting the ground truth relations to those involving entities that co-occur within the same sentence, as SemRep operates at the sentence level by default (SemRep-SENTENCE, 746 relationships).

To enable automatic evaluation on the CDR corpus, we mapped all MeSH identifiers in this corpus to UMLS CUIs using the UMLS REST API. As the relationships in the corpus are causal, we limited the evaluation to semantic predications with causal predicates: CAUSES, AFFECTS, AUGMENTS, STIMULATES, PREDISPOSES, and ASSOCIATED_WITH. We measured precision, recall, and F _1_ score. We assessed semantic predications using the following criteria:

- *True positive*: SemRep predication arguments match the chemical-disease pair with respect to CUI identifiers, UMLS preferred names, or mentions. If a predication argument is a more specific concept than the corresponding entity in the CDR corpus, this is also considered a match (e.g., the ground truth disease is *seizures* and the predication disease is *clonic seizures*).
- *False positive*: Predication arguments match entities in the ground truth but no relationship is annotated between the entities in the corpus. Another case is one in which a predication *contradicts* a ground truth relationship, i.e., predication arguments match those of a ground truth relationship, but the predicate type is an opposing relation type. Opposing types in this case are treats, prevents, in addition to the negated counterparts of the causal predicate types above [76].

All ground truth relationships without a matching predication are considered false negative instances.

The results of this evaluation are provided in Table 4, along with comparable results from the best-performing system in the BioCreative V CID task [19] as well as the state-of-the-art results [20]. Note that we limited the comparison to those systems that performed named entity recognition as well as relation extraction (i.e., end-to-end systems). Using SemRep 1.8, we achieved superior precision (0.90), at the expense of relatively low recall (0.24 with SemRep-ALL and 0.35 with SemRep-SENTENCE). SemRep does not attempt to recognize cross-sentence relationships; thus, the performance reported on the sentence-level evaluation (SemRep-SENTENCE) can be considered a fairer representation of its capabilities.

**Table 4:** Evaluation against the CDR corpus

	Precision	Recall	F _1_
SemRep-ALL	0.90	0.24	0.38
SemRep-SENTENCE	0.90	0.35	0.50
Xu et al. [19]	0.56	0.58	0.57
Peng et al. [20]	0.66	0.57	0.61

SemRep-ALL indicates the case in which all ground truth relations are taken into account. SemRep-SENTENCE indicates the scenario in which only the intra-sentence ground truth relations are considered. Xu et al. [19] was the top-ranking system in the BioCreative V CID task and Peng et al. [20] reported best post-challenge results. Both systems perform end-to-end relation extraction

## Discussion

### SemRep evaluation

Considering its breadth, SemRep provides reasonable precision on the test collection (0.69), while its recall is low (0.42), as is typical of rule-based systems. Error analysis revealed named entity recognition and normalization (NER) using MetaMap/UMLS as the single most problematic area in SemRep processing (26.9% of errors). This is not entirely surprising; in a recent evaluation [77], MetaMap yielded F _1_ scores in the range of 0.37-0.67 on various benchmark biomedical corpora. Limitations of MetaMap are compounded by the fact that the UMLS Metathaurus has been designed as a compendium of biomedical vocabularies, rather than a single, internally consistent terminology with a common architecture, rendering problematic its use as an terminological resource.

With respect to core aspects of SemRep processing (post-referential and relational analysis steps), the limitations of argument identification rules are the biggest source of errors (14%), followed by trigger detection errors (12.5%). In the absence of full dependency grammar, syntactic argument identification rules are underspecified and leave most of the heavy lifting to semantic constraints, which can fail in complex sentences containing multiple concepts of the same semantic group, leading to precision (type I) errors. Trigger detection errors, on the other hand, are mostly recall (type II) errors, indicating missing indicator rules. We note that some of these missing indicator rules had in fact been part of SemRep before, but have later been deactivated, as they led to too many false positives. This trade-off between precision and recall is an ongoing concern with SemRep. Prepositional indicators can be too ambiguous, and while recent enhancements [67] improved precision of predications generated by prepositional indicators, they still cause a significant number of errors.

Pre-processing (pre-linguistic and lexical/syntactic analysis steps) causes about 5% of the errors. A significant portion of these errors are due to part-of-speech disambiguation with the MedPost tagger, which was unexpected considering its restricted use in SemRep. A particular difficulty is the tagging of gerunds and participles, which can lead to errors in downstream shallow parsing, and in turn, referential and relational analysis. Shallow parsing per se did not cause as many errors as might have been expected (1.4%), suggesting that underspecified argument identification rules combined with semantic constraints compensate, to some extent, the lack of full constituent or dependency parsing in SemRep.

### Comparison to other relation extraction systems

Comparison of SemRep to other systems has been rare, primarily because there is no single relation extraction system targeting the UMLS domain knowledge with the same scope and coverage. A fair comparison requires adapting SemRep to task/corpus specifications or significant post-processing of its output. One notable exception was the evaluation of SemRep’s sortal anaphora resolution module on the BioNLP protein coreference dataset [78], which yielded results slightly better than the state-of-the-art results at the time [50].

In this study, we evaluated SemRep on the CDR corpus, a widely-used relation extraction benchmark. While precision was significantly higher than the reported best results on this corpus, recall lagged behind. Low recall was not surprising, as SemRep did not attempt to extract relations beyond sentences, which accounted for about 27% of all relations in the corpus. It is also important to note the several important differences between SemRep and the systems to which it was compared:

- SemRep was not trained on the CDR corpus or on any other weakly labeled data.
- These systems incorporate named entity recognizers also specifically trained on this corpus, which yield higher performance than MetaMap.
- High-performing systems use external knowledge base features that are highly predictive, such as those derived from Comparative Toxicogenomics Database which contains curated chemical-induced disease relationships.
- A significant portion of the relations in the CDR corpus are implicit, temporal inferences^3^, rather than explicit assertions [75], and SemRep’s inferencing machinery does not extend to such veiled inferences.

On the other hand, SemRep’s high precision on the corpus was state-of-the-art, and confirms that SemRep predications can be beneficial for this task as features or embeddings with high predictive value, as was explored to some extent previously by Pons et al. [79].

Most current relation extraction systems are based on machine learning models, trained and evaluated on standard benchmark corpora. Their generalizability to unseen relation and text types is generally found to be limited. While types of features used by systems trained on different corpora are generally similar, they often require retraining and fine-tuning to be successful on a different corpus [28]. Domain adaptation techniques have been applied to address this problem [17, 18, 80] with limited success, depending on the similarity of the source and target corpora. Given these issues and the difficulty of manually annotating corpora, it can be desirable to develop systems that can be generally applicable without much training data or customization. Even when such systems are less successful on a given benchmark corpus than models specifically trained on that corpus, they can still have great value as strong baseline systems, as demonstrated by MetaMap [41], one such system focusing on biomedical NER that has found widespread use. SemRep aims to serve as such a broad-coverage, strong baseline relation extraction system. SemRep also adopts an incremental development philosophy, allowing gradual improvements to the program. More importantly, its results are interpretable/explainable, because it is a rule-based system. This is unlike most machine learning approaches that produce black-box models, which is increasingly seen as a problem, particularly in the biomedical domain [81]. With these features and goals, SemRep stands apart from most biomedical relation extraction systems currently available. It is worth noting that some of the more successful systems that have been developed under DARPA’s recent Big Mechanism program [82], which focused on *machine reading* of full-text articles on cancer signaling pathways, have been rule-based and share similarities with SemRep. For example, TRIPS [24] is a deep semantic parser that uses syntactic, semantic, and ontological constraints and REACH [23] is a cascade of automata that relies on grammars to extract entities and events.

### Uses and Impact of SemRep

Despite its known limitations, SemRep has found widespread use in the scientific community. This has been facilitated primarily by SemMedDB [54], which provides a computable, semantic predication-based snapshot of the biomedical literature knowledge (essentially a massive knowledge graph), suitable for large-scale data mining and machine learning. SemRep has supported many tasks through SemMedDB, including identification of various types of biomedical associations (e.g., drug-drug interactions in clinical data [73], adverse drug reactions [83], chemical-disease relations [79], treatment/causation relations [84]), clinical decision making [85, 86], clinical guideline development [87], *in silico* screening for drug repurposing [88–90], gene regulatory network inference [91], biomedical question answering [74], elucidating gene-disease associations [92], medical diagnosis [93], link prediction [94], semantic relatedness assessment [95], and fact checking [96]. SemMedDB has also been used to generate new resources, including corpora (e.g., contradictions [76, 97], drug-drug interactions [98]), distributed representations of literature knowledge (i.e., embeddings) [99, 100], as well as vocabularies for alternative medicine therapies [101].

A research area that has particularly benefitted from SemRep/SemMedDB is literature-based discovery and hypothesis generation [93, 94, 102–115] (see Henry and McInnes [116] for a survey of this research area, including the use of SemRep/SemMedDB). An exciting recent development is the incorporation of SemMedDB into the Biomedical Data Translator platform [117], developed at the National Center for Advancing Translational Sciences (NCATS), which brings together disparate biomedical data sources (e.g., patient data, exposure data, biological pathways, literature) to support the translation of data into knowledge by applying automated reasoning methods to a graph representation of biomedical entities and their relationships. In one of its success stories, the platform was used to propose potential treatments for a five-year old patient with a rare genetic disorder, leading to significant improvement in his quality of life^4^.

### Future directions

The evaluation results presented in this paper inform our priorities and future directions, as we redesign SemRep as a more modular, flexible architecture and reimplement it in the Java programming language, which has the major advantage of allowing us to more easily incorporate third-party tools for specific tasks. For example, SemRep currently does not perform pronominal anaphora resolution, for which we presented a successful approach implemented in Java [118]. Similarly, a method for coordination ellipsis recognition and resolution [119] could be used to address this significant mapping problem. Furthermore, some third-party tools SemRep currently uses can be replaced by more recent state-of-the-art alternatives (e.g., GNormPlus [120] as a substitute for ABGene). Even more broadly, it becomes feasible to replace MetaMap with another NER tool that targets a specific domain when we process text in that domain. Comparison of SemRep to other systems on various tasks/corpora also becomes less of a challenge.

With the current availability and high performance of constituent and dependency parsers (e.g., Stanford CoreNLP [121]), an important question is whether SemRep should use such a parser instead of its shallow parsing approach, which could simplify some of the analysis steps at the expense of processing speed. However, we did not find evidence that the shallow parsing approach was a significant source of SemRep errors; therefore, we plan to continue using shallow parsing as the primary syntactic analysis approach. On the other hand, some rule-based systems incorporating dependency parsing with trigger detection and argument identification rules have yielded competitive performance in shared task competitions [21, 22], and we will consider incorporating dependency parsing as a processing option.

The prevalence of NER errors suggests that this mapping procedure needs closer scrutiny. By default, SemRep treats all vocabularies in the UMLS Metathesaurus the same way and prefers longest string matching. Earlier, we noted the problems with using the UMLS Metathesaurus as an terminological resource. Some research focusing on generating UMLS views for NLP [122] and community efforts like Open Biomedical Ontologies Foundry [123] aim to address these shortcomings of the UMLS. Almost all research in biomedical NER focuses on specific entity types (disorders, drugs, chemicals, etc.) and in benchmark corpora, entities are generally normalized to a single vocabulary/ontology (e.g., SNOMED CT [124] for disorders, NCBI Gene [58] for genes). This kind of selective use of the UMLS Metathesaurus vocabularies seems sensible and cleaner, given the interchangeable concepts and other issues we observed, and the additional processing we perform to mitigate these issues, such as dysonym processing. MetaMap already provides the ability to map only to specific vocabularies, and we will explore this option in more depth. Furthermore, given that SemRep does not generate predications involving some semantic types (e.g., Idea or Concept), it may be reasonable to invoke the semantic type selection option of MetaMap with SemRep.

Our evaluation also reveals shortcomings in our test collection, even when we put aside the annotation errors and its relatively small size. Relation annotation against the entire UMLS Metathesaurus is extremely difficult given its size (more than 4M concepts in the 2019AB release). This difficulty is exacerbated by the need to keep the test collection up-to-date with each UMLS release, which requires significant resources. A more reasonable evaluation approach for us could be to use benchmark relation extraction corpora, which are becoming increasingly common [1, 12]. This strategy is similar to the recent MetaMap evaluation strategy [77]. However, in contrast to NER corpora, relation corpora differ from each other and SemRep in their representation formalism, and not all map to the UMLS vocabularies, making this evaluation challenging. As we have shown with the evaluation on the CDR corpus, SemRep output needs to be tailored to some extent to make evaluation and comparison possible. The ability to map to non-UMLS vocabularies/ontologies can facilitate such evaluation. A MetaMap-related tool, Data File Builder [125], which allows building vocabularies from other resources, can be helpful in this regard.

SemRep development involves a significant amount of manual work in the form of linguistic analysis and refinement. Another future direction is to streamline this process and, to some extent, to semi-automate it. Automatic *ontology learning* [126] approaches can be used as the first step toward semi-automation. For example, keyphrase extraction techniques [127] can be used to identify concepts for specific domains using large-scale text corpora. New ontological predications and indicator rules can be learned based on concept-concept and concept-predicate co-occurrence patterns in corpora and statistical analysis. We plan to explore the use and expansion of another MetaMap-related tool, Custom Taxonomy Builder [128], to streamline these tasks.

Other research directions for SemRep include full-text processing and cross-sentence relation extraction. The former is largely a matter of building infrastructure, and potentially, refining some aspects of SemRep, such as sentence splitting, as full text articles exhibit structural differences from abstracts [129]. SemRep currently limits cross-sentence relation extraction to cases licensed by sortal anaphora resolution, but other types of discourse phenomena (e.g., document topic as implicit argument) also license such relations [75], and we plan to expand SemRep processing to consider such phenomena.

## Conclusions

We presented an in-depth description of SemRep and proposed it as a broad-coverage, high-performing baseline relation extraction system. Our depiction of SemRep in this paper is the most complete to date, and supersedes the more focused descriptions provided in earlier publications. Our evaluation provided a more accurate characterization of overall SemRep performance than those presented in prior evaluations. Our additional evaluation on a standard benchmark corpus confirmed its position as a strong baseline relation extraction system.

Through gradual improvements over time, SemRep has attained a level of maturity, with meaningful impact on clinical applications and biomedical research. While most users of SemRep choose the SemMedDB repository as the point of access, a command line version publicly available for Linux systems can also be used when documents of interest are not PubMed abstracts. For convenience, a web interface that can be used to process text interactively or in batch mode without installing the system is also provided (https://ii.nlm.nih.gov/Interactive/UTS_Required/semrep.shtml). A UMLS license is required to use SemRep.

Going forward, the incremental nature of SemRep development will allow us to address specific linguistic structures, relation types, and domains, as well as weaknesses identified through error analysis, while it remains strongly grounded in linguistic theory. We believe that this, combined with the fact that future development will take place in Java, a language more flexible and modular than Prolog, will enable us to improve SemRep performance and coverage more efficiently and increase its utility for clinical applications and biomedical discovery.

## Availability and requirements

**Project name:** SemRep **Project home page:**https://github.com/lhncbc/SemRep**Operating system(s):** Linux **Programming language:** SICStus Prolog with C/C++ extensions **Other requirements:** Approximately 60G disk space (assuming installation of all SemRep data files) **License:** UMLS license **Any restrictions to use by non-academics:** UMLS license needed

## Supplementary information

**Additional file 1** Appendix. A PDF file that contains illustration of SemRep processing steps on an example sentence from PubMed abstract 12975721. It also contains a detailed exposition of SemRep error analysis.

**Additional file 2** SemRep ontology. A text file that includes all SemanticType-predicate-SemanticType triples (*ontological predications*) used by SemRep.

**Additional file 3** SemRep indicator rules. A text file that includes all SemRep indicator rules, which are used to map textua expressions to semantic predicates.

## Data Availability

A Linux implementation of SemRep 1.8 is publicly available at https://semrep.nlm.nih.gov. SemRep test collection used for evaluation is available at https://semrep.nlm.nih.gov/GoldStandard.html. SemRep ontology and the indicator rules are made available as supplementary material.

## Abbreviations

CUI: Concept unique identifier
FN: False negative
FP: False positive
NER: Named entity recognition and normalization
NLP: Natural language processing
NP: Noun phrase
TP: True positive
UMLS: Unified Medical Language System

## Footnotes

1 Note that our definition of a noun phrase ignores concepts expressed over more complex, post-modified noun phrases, such as *pain in the leg*, which would be parsed as a noun phrase followed by a prepositional phrase.

2 It is worth noting that additional MetaMap options can be accommodated in SemRep using mm_add option, or the default MetaMap options can be turned off using mm_sub option, though SemRep in generic mode uses neither.

3 An example is the causal relationship between *methotrexate* and *acute renal failure* inferred from the sentence *Acute renal failure after high-dose methotrexate therapy in a patient with ileostomy.*

4 https://ncats.nih.gov/tidbit/tidbit_04.html
