# Supplementary material for: Broad-coverage biomedical relation extraction with SemRep
Source: BMC Bioinformatics. 2020 May 14;21:188. doi: 10.1186/s12859-020-3517-7 (PMC7222583; doi:10.1186/s12859-020-3517-7)
Supplement: Supplementary file 1 — Additional file 1 Appendix. A PDF file that contains illustration of SemRep processing steps on an example sentence from PubMed abstract 12975721. It also contains a detailed exposition of SemRep error analysis. [file 12859_2020_3517_MOESM1_ESM.pdf]

Appendix to
Broad-coverage biomedical relation extraction with SemRep

Halil Kilicoglu, Graciela Rosemblat, Marcelo Fiszman, and Dongwook Shin

February 21, 2020

Contents

S1. Illustration of the SemRep pipeline on an example sentence **2**

S2. SemRep error analysis **5**

S1. Illustration of the SemRep pipeline on an example sentence

We illustrate the steps of the SemRep pipeline on the following sentence, taken from the PubMed abstract 12975721. The results shown are obtained using SemRep 1.8 with the default options.

(1) *Low-dose diuretics, beta-blockers, angiotensin-converting enzyme inhibitors, and dihydropyridine calcium antagonists have reduced cardiovascular events in patients with diabetes.*

Pre-linguistic analysis Since we focus on a single sentence, sentence splitting is not shown. No acronym expansion is needed for this sentence. Below, we show the tokenization.
{`Low, -, dose, diuretics, beta, -, blockers, ,, angiotensin, -, converting, enzyme, inhibitors, ,, and, dihydropyridine, calcium, antagonists, have, reduced, cardiovascular, events, in, patients, with, diabetes`}.

Lexical/syntactic analysis The result of shallow parsing is shown below. Most of the lexical details (such as grammatical number, subcategorization frames) are omitted for readability. Each line represents a single syntactic unit (chunk). Simple noun phrases and prepositional phrases are shown as NP and PP, respectively.
[`mod(low-dose,noun), head(diuretics,noun), punc(,)`] (NP)
[`head(beta-blockers,noun), punc(,)`] (NP)
[`head(angiotensin-converting enzyme inhibitors,noun), punc(,)`] (NP)
[`conj(and,conj)`]
[`mod(dihydropyridine,noun), head(calcium antagonists,noun)`] (NP)
[`aux(have,aux)`]
[`verb(reduced,verb)`]
[`mod(cardiovascular,adj), head(events,noun)`] (NP)
[`prep(in,prep), head(patients,noun)`] (PP)
[`prep(with,prep), head(diabetes,noun), punc(.)`] (PP)

Referential analysis The following UMLS Metathesaurus concepts are identified using MetaMap. No NCBI Gene term is identified in this sentence.

- `C0445550: Low dose (Quantitative Concept)`
- `C0012798: Diuretics (Pharmacologic Substance)`
- `C0001645: Adrenergic beta-Antagonists (Pharmacologic Substance)`
- `C0003015: Angiotensin-Converting Enzyme Inhibitors (Pharmacologic Substance)`
- `C0220821: dihydropyridine (Pharmacologic Substance, Organic Chemical)`
- `C0006684: Calcium Channel Blockers (Pharmacologic Substance)`
- `C0392756: Reduced (Qualitative Concept)`
- `C1320716: Cardiovascular event (Finding)`
- `C0030705: Patients (Patient or Disable Group)`
- `C0011847: Diabetes (Disease or Syndrome)`

Post-referential analysis No empty heads are marked in this sentence. The noun phrase coordination module identifies *low-dose diuretics*, *beta-blockers*, *angiotensin-converting enzyme inhibitors*, and *dihydropyridine calcium antagonists* as conjuncts in a series coordination, because they are semantically compatible (they all belong to the `Drugs & Chemicals` semantic group), and are separated only by commas and the coordinating conjunction *and*.

Relational analysis Hypernymy processing identifies the following hypernymic predication, due to the presence of nominal modification structure (*dihydropyridine calcium antagonists*) and the semantic compatibility of concepts.

- `C0220821: dihydropyridine (Pharmacologic Substance, Organic Chemical)` - ISA -
  `C0006684: Calcium Channel Blockers (Pharmacologic Substance)`

The following predications are identified due to verbal argument identification rules; for the first, *cardiovascular events* is recognized as the semantic object of the verb *reduced* and *calcium antagonists* as the semantic subject. This predication is supported by the indicator rule `reduce:verb:none` → INHIBITS and the ontological predication `Pharmacologic Substance`-INHIBITS-`Finding`. Since *calcium antagonists* is coordinated with *low-dose diuretics*, *beta-blockers*, and *angiotensin-converting enzyme inhibitors*, the next three predications are also generated. This illustrates the reuse of the object argument (`Cardiovascular event`) due to noun phrase coordination.

- `C0006684: Calcium Channel Blockers (Pharmacologic Substance)` - INHIBITS -
  `C1320716: Cardiovascular event (Finding)`
- `C0012798: Diuretics (Pharmacologic Substance)` - INHIBITS -
  `C1320716: Cardiovascular event (Finding)`
- `C0001645: Adrenergic beta-Antagonists (Pharmacologic Substance)` - INHIBITS -
  `C1320716: Cardiovascular event (Finding)`
- `C0003015: Angiotensin-Converting Enzyme Inhibitors (Pharmacologic Substance)` - INHIBITS -
  `C1320716: Cardiovascular event (Finding)`

Two predications below are identified using prepositional argument identification rules. The first is supported by the indicator rule `in:prep:none` → PROCESS_OF and the second one by the rule `with:prep:none` → HAS_PROCESS. Ontological predications relevant to the extracted SemRep predications are `Disease or Syndrome`-PROCESS_OF-`Human` and `Finding`-PROCESS_OF-`Human`, respectively.

- `C0011847: Diabetes (Disease or Syndrome)` - PROCESS_OF -
  `C0030705: Patients (Patient or Disabled Group, Human)`
- `C1320716: Cardiovascular event (Finding)` - PROCESS_OF -
  `C0030705: Patients (Patient or Disabled Group, Human)`

Finally, the following predication is generated via the inferencing rule `IF <X-anyRelation-Y AND Z-ISA-Y> THEN <Z-anyRelation-Y>`.

- `C0220821: dihydropyridine (Pharmacologic Substance, Organic Chemical)` - INHIBITS (INFER) -
  `C1320716: Cardiovascular event (Finding)`

The comparative processing and negation processing steps are not called for in this sentence.

The sentence and all extracted predications are repeated below.

Low-dose diuretics, beta-blockers, angiotensin-converting enzyme inhibitors, and dihydropyridine calcium antagonists have reduced cardiovascular events in patients with diabetes.

- `C0220821: dihydropyridine (Pharmacologic Substance, Organic Chemical)` - ISA -
  `C0006684: Calcium Channel Blockers (Pharmacologic Substance)`
- `C0006684: Calcium Channel Blockers (Pharmacologic Substance)` - INHIBITS -
  `C1320716: Cardiovascular event (Finding)`
- `C0012798: Diuretics (Pharmacologic Substance)` - INHIBITS -
  `C1320716: Cardiovascular event (Finding)`
- `C0001645: Adrenergic beta-Antagonists (Pharmacologic Substance)` - INHIBITS -
  `C1320716: Cardiovascular event (Finding)`
- `C0003015: Angiotensin-Converting Enzyme Inhibitors (Pharmacologic Substance)` - INHIBITS -
  `C1320716: Cardiovascular event (Finding)`
- `C0011847: Diabetes (Disease or Syndrome)` - PROCESS_OF -
  `C0030705: Patients (Patient or Disabled Group, Human)`
- `C1320716: Cardiovascular event (Finding)` - PROCESS_OF -
  `C0030705: Patients (Patient or Disabled Group, Human)`
- `C0220821: dihydropyridine (Pharmacologic Substance, Organic Chemical)` - INHIBITS (INFER) -
  `C1320716: Cardiovascular event (Finding)`

S2. SemRep error analysis

Two authors (GR and HK) carried out an error analysis of SemRep, using the results obtained on the SemRep test collection. We first independently analyzed errors from 20 abstracts and developed a categorization of errors, aligning it with the SemRep processing steps. After discussing the errors on these 20 abstracts, reconciling the differences, and refining the categorization, GR carried out the rest of the error analysis. HK finalized the analysis by making judgments on the challenging cases. Inter-annotator agreement was calculated on the first 20 abstracts (Cohen's κ=0.84, considered almost perfect agreement). Since SemRep has a pipeline architecture, it is possible that an error may be due to several factors, and even if one cause is addressed, another unrelated error could still be triggered. For this reason, we attempted to identify multiple causes for an error, when possible. The resulting categorization of errors and their distribution is given in Figure 1.

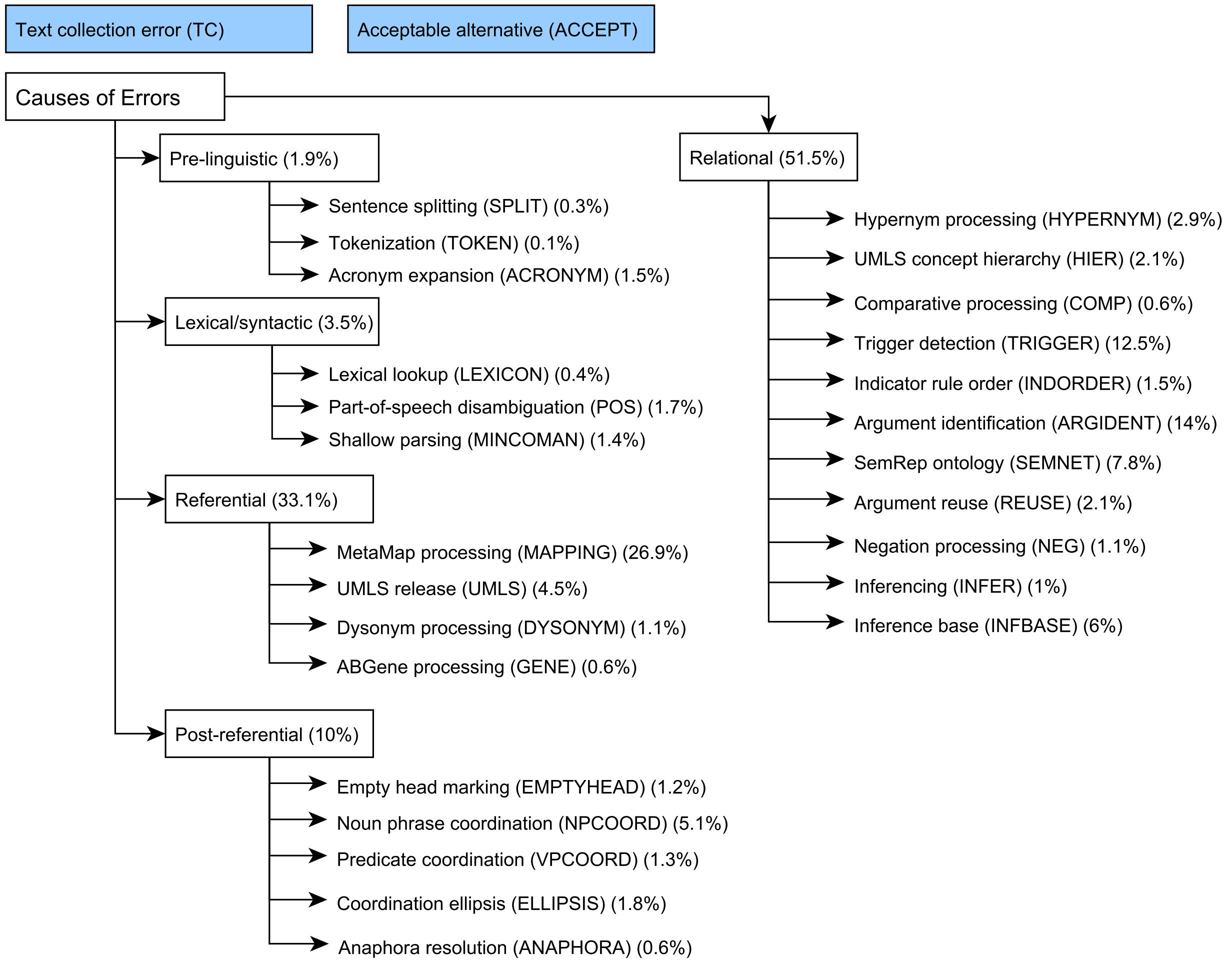

Figure 1: A categorization of SemRep errors. Blue boxes represent categories that are considered errors only in strict evaluation.

The first two categories (shown in blue in Figure 1) are not actual errors, but point to issues with the test collection and the strict evaluation. Relaxed evaluation addresses these issues, resulting in better performance (about 24% improvement in F_1 score).

Test collection error (TC) These are cases in which we found that a predication marked as false positive (FP) was in fact acceptable. In the example below, the hypernymic predication identified by SemRep had not been annotated in the test collection.

(2) *... to conduct a meta-analysis comparing the efficacy of rabeprazole and other proton pump inhibitors when co-prescribed with antibiotics.*
`Rabeprazole`-ISA-`Proton Pump Inhibitor` (FP)

In a smaller number of cases, we also found that the annotated predication was not a good semantic representation of the meaning of the sentence. In the example below, we considered `genetic aspects (Biologic Function)` a bad mapping for *genetics*.

(3) *The genetics of ray pattern variation in Caenorhabditis briggsae.*
`genetic aspects`-PROCESS_OF-`Caenorhabditis briggsae` (FN)

Acceptable alternative (ACCEPT) These are cases in which two concepts, one identified by SemRep and the other annotated in the test collection, are interchangeable and therefore the generated predication is acceptable. However, because strict evaluation uses CUIs or NCBI Gene IDs for concept matching, these were evaluated as incorrect. In the example below, concepts `Human` and `Homo sapiens` are considered interchangeable. These cases point to the limitation of using the UMLS Metathesaurus as an ontological resource, in the presence of considerable overlap between concepts.

(4) *ELISA and immunoblotting using glycoproteins purified by preparative isoelectric focusing were used to detect humancysticercosis...*
`Cysticercosis`-PROCESS_OF-`Human` (FP)
`Cysticercosis`-PROCESS_OF-`Homo sapiens` (FN)

We present errors generated by specific SemRep components below. The frequency of the error types in our evaluation is indicated in parentheses.

Sentence splitting (SPLIT) (0.3%) Incorrect sentence splittings may lead to over-generation of predications, or to missing predications. In the example below, the percentage sign is not recognized as a sentence boundary, leading to four false positive predications, triggered by *risk*.

(5) *A risk assessment model that included familial risk, demographics, and personal history of diabetes, hypercholesterolemia, hypertension, and obesity was most optimal with an area under the curve statistic of 87.2% CONCLUSIONS: Familial risk assessment can stratify* ***risk*** *for early-onset coronary heart disease.*

- `Diabetes`-PREDISPOSES-`Coronary heart disease` (FP)
- `Hypertensive disease`-PREDISPOSES-`Coronary heart disease` (FP)
- `Obesity`-PREDISPOSES-`Coronary heart disease` (FP)
- `hypercholesterolemia`-PREDISPOSES-`Coronary heart disease` (FP)

Tokenization (TOKEN) (0.1%) In the example below, *poly-(radiculo)neuropathy* is tokenized as [*poly,-,(,radiculo,),neuropathy*], rather than being treated as a single unit, leading to the incorrect hypernymic predication (which is technically correct, but should not have been generated from this sentence).

(6) *Guillain-Barre syndrome (GBS) and chronic inflammatory demyelinating poly-(radiculo)neuropathy (CIDP) are immune-mediated disorders with a variable duration of progression and a range in severity of weakness.*

- `Polyradiculoneuropathy, Chronic Inflammatory Demyelinating`-ISA-`Neuropathy` (FP)

Acronym expansion (ACRONYM) (1.5%) Lack of acronym expansion may cause SemRep to miss legitimate predications. In the following, *OVX* was not expanded to *ovariectomy* when it was first used in the abstract (*ovariectomized (OVX) rats*), resulting in a false negative predication.

(7) *In conclusion, the AM extract produced a very weak effect on the prevention of bone loss* ***induced*** *by OVX and Ca deficiency in rats, but was similar to the results observed with alendronate.*

- `Ovariectomy`-CAUSES-`loss; bone` (FN)

Lexical lookup (LEXICON) (0.4%) An entry in the SPECIALIST Lexicon can interfere with later steps in SemRep. This is generally due to multi-word expressions in the Lexicon. Below, the fact that *oral administration* is treated as a single lexical unit (i.e., it is a multi-word expression in Lexicon) and we have an indicator rule involving *administration*, but not *oral administration*, prevents us from extracting the predication shown.

(8) *... oral* ***administration*** *of dibutyl phthalate (DBP) to female Long Evans (LE) hooded rats from weaning through puberty, mating, and gestation disrupts pregnancy maintenance ...*

- `Dibutyl Phthalate`-ADMINISTERED_TO-`Rats, Long-Evans` (FN)

Part-of-speech disambiguation (POS) (1.7%) POS tags returned from the Lexicon are disambiguated by the MedPost tagger. The Lexicon tends to include many abbreviations and other biomedicine-specific terms, which MedPost can disambiguate incorrectly. In the example, the preposition *via* is ambiguous between noun and preposition (the noun entry corresponding to the acronym of *video image analysis*). MedPost disambiguates it as noun, leading to false negatives.

(9) *Rho signaling via ROCK has been previously shown either to* ***activate*** *or to* ***downregulate*** *PI3K/Akt.*

- `rho`-INHIBITS-`1-Phosphatidylinositol 3-Kinase` (FN)
- `rho`-INHIBITS-`Proto-Oncogene Proteins c-akt` (FN)
- `rho`-STIMULATES-`1-Phosphatidylinositol 3-Kinase` (FN)
- `rho`-STIMULATES-`Proto-Oncogene Proteins c-akt` (FN)

Shallow parsing (MINCOMAN) (1.4%) Shallow parsing errors can be caused by a myriad of reasons. In the example below, acronym expansion is unable to expand *BBB* to *blood-brain barrier*; therefore, *blood-brain barrier permeability* is not recognized as a single NP, and the predication below is missed.

(10) *We have recently shown that melatonin decreases the late (24 hr) increase in blood-brain barrier (BBB) permeability and the risk of tissue plasminogen activator-induced hemorrhagic transformation following ischemic stroke in mice.*

- `Permeability`-PROCESS_OF-`Blood - brain barrier anatomy` (FN)

MetaMap processing (MAPPING) (26.9%) MetaMap processing issues account for most errors in SemRep. Preference for longer matches in mapping is the cause of the missed predication in the first example below. The phrase *large tumor* is mapped to the concept `Large Tumor (Qualitative Concept)`, instead of the more appropriate `Neoplasms (Neoplastic Process)`, and subsequently, the lack of an ontological predication `Body Location or Region`-LOCATION_OF-`Qualitative Concept` means we miss the given predication.

(11) *An unusual case of 4-year old girl presenting large tumor* ***of*** *the neck with massive calcification is described.*

- `Neck`-LOCATION_OF-`Tumor` (FN)

In the following example, lack of coordination ellipsis resolution (i.e., resolution of *removal and reinsertion of peritoneal dialysis catheter* into *removal of peritoneal dialysis catheter* and *reinsertion of peritoneal dialysis catheter*) leads to both a precision and a recall error.

(12) ***Treatment*** *of refractory pseudomonas aeruginosa exit-site infection by simultaneous removal and reinsertion of peritoneal dialysis catheter.*

- `Surgical Replantation`-TREATS-`Pseudomonas aeruginosa infection NOS` (FP)
- `Insertion of temporary peritoneal dialysis catheter`-TREATS-`Pseudomonas aeruginosa infection NOS` (FN)

UMLS release (UMLS) (4.5%) These errors are due to using UMLS 2006AA as the default release for SemRep, instead of the latest. In the following example, SemRep extracts `subject (Idea or Concept)` for *subjects* instead of `Human Study Subject (Human)` and `heme-1 (Organic Chemical)` for *VAP-1* instead of `AOC3 protein, human (Amino Acid, Peptide, or Protein)`, leading to two false negatives. When running SemRep with UMLS 2018AA release, we get both predications.

(13) *Clinical characteristics and serum VAP-1 levels **in** 20 morbidly obese subjects (mean BMI 38.84 kg/m(2)) were measured ...*

- `Obesity`-PROCESS_OF-`Human Study Subject` (FN)
- `AOC3 protein, human`-PART_OF-`Human Study Subject` (FN)

Dysonym processing (DYSONYM) (1.1%) Substring matching rules in dysonym processing have some shortcomings, manifesting themselves as missed concepts and subsequently, missed predications. In the example below, *pacemaker* is originally mapped to `Artificial cardiac pacemaker (Medical Device)`, but this mapping is removed because it is considered a dysonym. Without the default dysonym processing option, this predication is generated.

(14) *Is a dual-sensor pacemaker appropriate **in** patients with sino-atrial disease?*

- `Artificial cardiac pacemaker`-TREATS-`Patients` (FN)

ABGene processing (GENE) (0.6%) ABGene can recognize a non-gene term as a gene, potentially triggering a FP, or miss a gene term, resulting in a FN. In the example below, *BMD* (an acronym for *bone mineral density*) is never expanded in the abstract, and is recognized as a gene mention by ABGene. Gene/protein normalization process then maps it to NCBI Gene terms `DMD` and `BEST1`, and a false positive predication is generated.

(15) *To assess the genetic and environmental determinants of BMD **in** southern Chinese women ...*

- `Woman`-LOCATION_OF-`DMD,BEST1` (FP)

Empty head marking (EMPTYHEAD) (1.2%) When terms marked as empty heads are ignored in semantic interpretation, incorrect predications can be generated due to the use of modifiers. In the example below, *concentrations* is marked as an empty head, and its modifier, *sex hormone*, is used for interpretation, leading to an incorrect predication.

(16) *Sex hormone concentrations **in** patients with rheumatoid arthritis*

- `Gonadal Steroid Hormones`-TREATS-`Patients` (FP)

Noun phrase coordination (NPCOORD) (5.1%) When SemRep is unable to detect noun phrase coordination, it can lead to recall errors. In the example below, NP coordination involving *bezafibrate*, *ibuprofen*, and *nitrazepam* is missed, due to the intervening parenthetical *(both R- and S-isomers)*. While two true positive predications are generated (also shown below), another four are missed as a result.

(17) *Herein we describe the **binding** of three structurally diverse lipophilic drugs, bezafibrate, ibuprofen (both R- and S-isomers) and nitrazepam to I-FABP.*

- `Nitrazepam`-INTERACTS_WITH-`Fatty Acid-Binding Proteins, Intestinal-Specific` (TP)
- `Bezafibrate`-ISA-`Pharmaceutical Preparations` (TP)
- `Ibuprofen`-INTERACTS_WITH-`Fatty Acid-Binding Proteins, Intestinal-Specific` (FN)
- `Bezafibrate`-INTERACTS_WITH-`Fatty Acid-Binding Proteins, Intestinal-Specific` (FN)
- `Ibuprofen`-ISA-`Pharmaceutical Preparations` (FN)
- `Nitrazepam`-ISA-`Pharmaceutical Preparations` (FN)

Predicate coordination (VPCOORD) (1.3%) SemRep's predicate coordination machinery is very limited, and this shortcoming can lead to recall errors. In the example below, while we identify one correct predication (shown below), another is missed because we cannot identify the coordination between the clauses *is neuroprotective ...* and *downregulates the dopamine transporter ...*.

(18) *Low dose pramipexole is* ***neuroprotective*** *in the MPTP mouse model of Parkinson's disease, and* ***downregulates*** *the dopamine transporter via the D3 receptor.*

- `pramipexol-PREVENTS-Parkinson Disease` (TP)
- `pramipexol-INHIBITS-dopamine transporter` (FN)

Coordination ellipsis (ELLIPSIS) (1.8%) This class of errors can also be attributed to shortcomings of MetaMap parsing, as they ultimately relate to mapping. They are due to the inability to identify the modifier coordination and expand it to generate the correct concept mappings. In the example below, *CD4+ and CD8+ T lymphocytes* cannot be expanded to *CD4+ T lymphocytes* and *CD8+ T lymphocytes*, and therefore, two mappings and subsequently two predications are missed. Note that a false positive is generated, as well.

(19) *Effects of human soluble BAFF synthesized in Escherichia coli on CD4+ and CD8+ T lymphocytes as well as NK cells* ***in*** *mice.*

- `Mus-LOCATION_OF-CD4 Gene` (FP)
- `Mus-LOCATION_OF-CD4 Positive T Lymphocytes` (FN)
- `Mus-LOCATION_OF-CD8-Positive T-Lymphocytes` (FN)

Anaphora resolution (ANAPHORA) (0.6%) SemRep does not perform pronominal anaphora resolution, and this can lead to problems in argument identification. In the following example, the possessive pronoun *their* refers to *Y(2) receptors*, but without this resolution, SemRep is unable to recognize that it is the semantic subject of the predicate *activation*.

(20) *The enhanced veratridine response observed in +/+ tissue following BIIE0246, indicates that Y(2) receptors are located on submucosal neurons and that their activation by NPY will* ***inhibit*** *enteric noncholinergic secretory neurotransmission.*

- `neuropeptide Y2 receptor-INHIBITS-Neuronal Transmission` (FN)

Hypernym processing (HYPERNYM) (2.9%) Hypernym resolution rules may be unable to deal with some syntactic complexities. In the following example, *is* is recognized as a trigger for a hypernymic predication, while the actual trigger for the predication between the concepts `Neurocysticercosis` and `nervous system disorder` is *cause*. Because a hypernymic predication is generated, SemRep is unable to generate the causal relationship.

(21) *Neurocysticercosis (NCC) is one of the major* ***causes*** *of neurological disease in China.*

- `Neurocysticercosis-ISA-nervous system disorder` (FP)
- `Neurocysticercosis-CAUSES-nervous system disorder` (FN)

UMLS concept hierarchy (HIER) (2.1%) Some legitimate hypernymic predications are missed because there is no hierarchical relationship between the concepts in the UMLS Metathesaurus. In the example, a hierarchical relationship cannot be found in the UMLS between `Antibiotics` and `Intervention regimes`. Several other predications are also missed, because the complex coordination of interventions cannot be detected.

(22) *The interventions investigated were: antibiotics (4); home versus hospital administration of antibiotics (1); steroids (1); mucolytic therapies (6); exercise (3) and pancreatic enzymes (1).*

- `Antibiotics-ISA-Intervention regimes` (FN)

Comparative processing (COMP) (0.6%) Comparative patterns used by SemRep can be too strict. In the example, the algorithm requires a noun phrase after *compared with* and the comparative trigger *as compared with that of* is not recognized, leading to a recall error.

(23) *The relative effectiveness of second-generation (atypical) antipsychotic drugs* ***as compared with that of*** *older agents has been incompletely addressed, though newer agents are currently used far more commonly.*

- `Antipsychotic Agents`-COMPARED_WITH-`Agent` (FN)

Trigger detection (TRIGGER) (12.5%) Lack of triggers for specific predicates can cause recall errors. In the example, the preposition *for* should trigger a DIAGNOSES predicate, but we don't have a corresponding indicator rule and miss the predication shown.

(24) *The current study assessed screening and preventive behaviors during 12 months after predictive genetic testing* ***for*** *hereditary nonpolyposis colorectal carcinoma (HNPCC) in an Australian clinical cohort.*

- `Testing, Genetic Predisposition`-DIAGNOSES-`Hereditary Nonpolyposis Colorectal Neoplasms` (FN)

Indicator rule order (INDORDER) (1.5%) Some triggers are ambiguous between different predicates, and the order in which they are applied can cause errors. In the example below, the preposition *in* can indicate either a TREATS or a LOCATION_OF predication. The indicator rule corresponding to TREATS is applied first, leading to a false positive and a false negative error.

(25) *Serum levels of DHEAS and free testosterone were markedly lower at baseline* ***in*** *patients*

- `Free testosterone`-TREATS-`Patients` (FP)
- `Free testosterone`-LOCATION_OF-`Patients` (FN)

Argument identification (ARGIDENT) (14%) Syntactic constraints on argument identification are underspecified and can lead to errors, especially when multiple concepts with the same semantic type occur in the same sentence and thus, semantic constraints are less effective. In the example below, the semantic subject of the trigger *detect* is recognized as *isoelectric focusing* instead of *immunoblotting*, since it is closer to the verbal trigger and it satisfies the semantic constraint for the predication. This leads to a false positive error, and two false negative errors (due to coordination).

(26) *ELISA and immunoblotting using glycoproteins purified by preparative isoelectric focusing were used to* ***detect*** *human cysticercosis in Tongliao area, Inner Mongolia, China in 1998.*

- `Isoelectric Focusing`-DIAGNOSES-`Cysticercosis` (FP)
- `Immunoblotting`-DIAGNOSES-`Cysticercosis` (FN)
- `Enzyme-Linked Immunosorbent Assay`-DIAGNOSES-`Cysticercosis` (FN)

SemRep ontology (SEMNET) (7.8%) Lack of an ontological predication in the SemRep ontology and presence of one that is not generally applicable can directly result in errors. In the example below, we do not have an ontological predication `Amino Acid, Peptide, or Protein`-AFFECTS-`Mental Process`, leading to two false negative errors.

(27) *The dopamine D3 receptor, which is highly enriched in nucleus accumbens (NAc), has been suggested to* ***play an important role*** *in reinforcement and reward.*

- `dopamine D3 receptor`-AFFECTS-`Rewards` (FN)
- `dopamine D3 receptor`-AFFECTS-`Psychological reinforcement` (FN)

Argument reuse (REUSE) (2.1%) Argument reuse rules rely on the detection of predicate coordination and relative clauses, but SemRep's machinery for these phenomena have shortcomings, generally leading to false negative errors. Below, the reuse of *implantation* in the second predication is blocked due to argument reuse rules.

(28) *The factors associated with recurrent restenosis* ***after*** *SES implantation* ***for*** *in-stent restenosis are unknown.*

- `Implantation procedure`-PRECEDES-`Restenosis` (TP)
- `Implantation procedure`-TREATS-`Restenosis` (FN)

Negation processing (NEG) (1.1%) Inability to recognize the negation of an argument or the predicate can lead to both precision and recall errors. In the example, the distance (in terms of chunks) between the trigger (*treatment*) and the negation trigger *not* is higher than what is allowed for negation to take effect, so we get two errors, shown below.

(29) *ESWL should* ***not*** *be considered as the first line of* ***treatment*** *in the morbidly obese patients.*

- `Extracorporeal Shockwave Lithotripsy`-TREATS-`Patients` (FP)
- `Extracorporeal Shockwave Lithotripsy`-NEG_TREATS-`Patients` (FN)

Inferencing (INFER) (1%) Some valid inferencing rules are not implemented, because they may not be broadly applicable. For the following example, we do not have the inference rule IF <X-PROCESS_OF-Y AND Z-PROCESS_OF-Y> THEN <Z-COEXISTS_WITH-X>, so we miss the predication shown, even though both base predications were generated.

(30) *Both sequential treatments can be safely performed for SCLC patients* ***with*** *brain metastases, may relieve neurological symptoms, and well control both primary and metastatic lesions.*

- `Small cell carcinoma of lung`-PROCESS_OF-`Patients` (TP)
- `Metastatic malignant neoplasm to brain`-PROCESS_OF-`Patients` (TP)
- `Metastatic malignant neoplasm to brain`-COEXISTS_WITH-`Small cell carcinoma of lung` (FN)

Inference base (INFBASE) (6%) It is not uncommon to generate an INFER predication when a false positive error has been generated due to one of the error types above. In the following example, the empty head marking problem led to a false positive error, which in turn led to an inference error.

(31) *Sex hormone concentrations* ***in*** *patients* ***with*** *rheumatoid arthritis are not normalized during 12 weeks of anti-tumor necrosis factor therapy.*

- `Rheumatoid arthritis`-PROCESS_OF-`Patients` (TP)
- `Gonadal Steroid Hormones`-TREATS-`Patients` (FP)
- `Gonadal Steroid Hormones`-TREATS(INFER)-`Rheumatoid arthritis` (FP)
